# Construction of diverse water wave structures for coupled nonlinear fractional Drinfel’d-Sokolov-Wilson model with Beta derivative and its modulus instability

**DOI:** 10.1038/s41598-023-44428-5

**Published:** 2023-10-16

**Authors:** Muhammad Shakeel, Salman A. AlQahtani, Muhammad Junaid U Rehman, Grzegorz Kudra, Jan Awrejcewicz, Abdulaziz M. Alawwad, Abdullilah A. Alotaibi, Mejdl Safran

**Affiliations:** 1 Department of Mathematics, Quaid-I-Azam University, Islamabad, Pakistan; 2 School of Mathematics and Statistics, Central South University, Changsha, 410083 China; 3 New Emerging Technologies and 5G Network and Beyond Research Chair, Department of Computer Engineering, College of Computer and Information Sciences, King Saud University, Riyadh, Saudi Arabia; 4 Department of Automation, Biomechanics, and Mechatronics, Lodz University of Technology, 1/15 Stefanowski St. (Building A22), Lodz, 90-924 Poland; 5 Department of Computer Science, College of Computer and Information Sciences, King Saud University, Riyadh, Saudi Arabia

**Keywords:** Engineering, Mathematics and computing, Physics

## Abstract

This paper aims to analyze the coupled nonlinear fractional Drinfel’d-Sokolov-Wilson (FDSW) model with beta derivative. The nonlinear FDSW equation plays an important role in describing dispersive water wave structures in mathematical physics and engineering, which is used to describe nonlinear surface gravity waves propagating over horizontal sea bed. We have applied the travelling wave transformation that converts the FDSW model to nonlinear ordinary differential equations. After that, we applied the generalized rational exponential function method (GERFM). Diverse types of soliton solution structures in the form of singular bright, periodic, dark, bell-shaped and trigonometric functions are attained via the proposed method. By selecting a suitable parametric value, the 3D, 2D and contour plots for some solutions are also displayed to visualize their nature in a better way. The modulation instability for the model is also discussed. The results show that the presented method is simple and powerful to get a novel soliton solution for nonlinear PDEs.

## Introduction

A solitary wave is a special type of wave that maintains its shape as it propagates through a medium, without changing its speed or amplitude. Solitary waves can arise in various fields, including water waves, metamaterials, engineering, plasma waves, and optical fibers^1–12^. In recent years, there has been increasing interest in the study of solitary waves in nonlinear fractional differential equations (NFDEs), which are differential equations involving fractional derivatives. NFDEs are generalizations of classical differential equations, in which the order of the derivative is not necessarily an integer. Solitary wave solutions of NFDEs have important applications in various fields, including physics, mathematics, engineering, and biology^13–20^. The study of solitary waves in NFDEs is a challenging task, due to the nonlinearity and fractional nature of these equations.

In recent few decades, many efficient methods or techniques have been used to find the analytical solutions for nonlinear models, such as the Ricatti approach^21^, the Kudryashov method^22^, the Darboux transformation^23^, the Jacobi elliptic function approach^24^, the sine-cosine approach^25^, the direct algebraic technique^26^, the extended tanh function method^27–31^, sine-Gordon approach^32,33^, Fokas technique^34^, the Hirota bilinear transformation approach^35,36^, the first integral approach^37^, the trial solution technique^38^, the $\left( \frac{G^{'}}{G}\right)$-expansion approach^39^, $\left( \frac{G^{'}}{G^2}\right)$-expansion technique^40^,$\left( \frac{G^{'}}{G},\frac{1}{G}\right)$-expansion technique^41–43^, Lie Symmetry method^44^, the unified method^45^, and so on. The travelling wave solution of DSW was attained by utilizing the auxiliary equation method^46^. By utilizing the modified extended direct algebraic method bell, anti-bell, periodic and dark solitary wave solution of DSW has been attained in^47^. The series solution of the DSW model was attained by using the Adomian decomposition method^48^.

The coupled (1+1)-dimensional DSW model^49^ which read as,

1 $$\begin{aligned} \begin{aligned} \Phi _t+a \Psi \Psi _x&=0\\ \Psi _t+\gamma _1\Psi \Phi _x+\lambda _1\Phi \Psi _x+\eta _1\Psi _{xxx}&=0. \end{aligned} \end{aligned}$$

We can write the above system in the form of fractional derivative with respect to time is given by,

2 $$\begin{aligned} \begin{aligned} D^\alpha _t\Phi +a \Psi D_x\Psi&=0\\ D^\alpha _t\Psi +\gamma _1\Psi D_x\Phi +\lambda _1\Phi D_x\Psi +\eta _1 D_{xxx}\Psi&=0. \end{aligned} \end{aligned}$$

Here, $a, \gamma _1, \lambda _1$ and $\eta _1$ are the constant and the $\alpha$ represents the order of fractional derivative with $0<\alpha \le 1$. When $\alpha =1$ Eq. (2) is converted to classical DSW equation, which was first introduced by Drinfel’d and Sokolov^50,51^ and studied by Wilson^52^. In this article, we will construct an exact solution for the Drinfel’d-Sokolov-Wilson model using the generalized rational exponential function method approach with the help of well-known Beta derivative. The solutions are attained in the form of singular bright, dark, periodic, bell and lump-type water wave structures. The achieved solutions might be useful to comprehend nonlinear phenomena. It is worth noting that the implemented method for solving NPDEs is efficient, and simple to find further and new-fangled solutions in the area of mathematical physics and coastal engineering. Diverse types of fractional derivatives have been used in the past, such as Caputo fractional^53^, Beta derivative^54^, Conformable fractional^55^, Reimann-Liouville^56^ and truncated M-fractional derivative^57^ etc. have importance in fractional calculus.

The remaining article is distributed into various sections. Section (2) contain definition from fractional calculus relevant to our study. In Sect. (3) we have discussed the main step of the method. In Sect. (4) solitary wave solutions have been described. Numerical simulations of some attained solutions are given in (5). In Sects. (6) and (7) modulus instability, a conclusion is presented.

## Beta derivative

### Definition

Let $\Pi (t)$ be a function defined for all non-negative *t*. The function $\Pi (t)$^58^ is,

3 $$\begin{aligned} D_t^\alpha \{\Pi (t)\}=\lim _{\varepsilon \rightarrow 0}{\frac{\Pi (t+\epsilon (t+\frac{1}{\Gamma (\alpha )})^{1-\alpha })-\Pi (t)}{\varepsilon }}, \end{aligned}$$

### Theorem

Let $\Pi$ and *g* be any two function, $\Pi \ne 0$, and $\alpha \in (0,1]$ then

**1:** $$D_t^\alpha \{b_1\Pi (t)+b_2\Upsilon (t)\}=b_1D_t^\alpha \Pi (t)+b_2D_t^\alpha \Upsilon (t)$$,

where $$b_1,b_2\in \Re$$

**2:** $$D_t^\alpha \{\Pi (t).\Upsilon (t)\}=\Pi (t)D_t^\alpha \{\Upsilon (t)\}+\Upsilon (t)D_t^\alpha \{\Pi (t)\}$$,

**3:** For *c* any constant, the following relation can be easily satisfied $D_t^\alpha c=0,$

**4:** $$D_t^\alpha (\frac{\Pi (t)}{\Upsilon (t)})=\frac{\Upsilon (t) D_t^\alpha \{\Pi (t)\}-\Pi (t) D_t^\alpha \{\Upsilon (t)\}}{\Upsilon (t)^2}$$,

**5:** $$D_t^\alpha \{\Pi (t)\}=(t+\frac{1}{\Gamma (\alpha )})^{1-\alpha }\frac{d\Pi (t)}{dt}$$,

## Methodology

The GERF method is a quite novel technique for nonlinear partial differential equations (NLPDE)^49^. The main steps are given as:

**Step:1**

Consider the NLPDE as,

4 $$\begin{aligned} H(\Omega , \Omega _x, \Omega _t, \Omega _{xx}, \Omega _{tt}...)=0. \end{aligned}$$

Suppose the travelling wave transformation,

5 $$\begin{aligned} \Omega (x,t)=\Psi (\varpi )e^{\iota \phi (x,t)}. \end{aligned}$$

Substituting (5) into (4) then we get ODE given as,

6 $$\begin{aligned} \digamma (\Psi , \Psi ^{'}, \Psi ^{''}, \Psi ^{'''},...)=0. \end{aligned}$$

**Step:2**

Solution of equation of (7) is,

7 $$\begin{aligned} \Psi (\varpi )=a_0+\sum _{n=1}^{N}(a_n\phi (\varpi )^{n}+b_n\phi (\varpi )^{-n}). \end{aligned}$$

Here, $a_0, a_{n}$, and $b_n$ are unknown parameters to be found. The function $\phi (\varpi )$ is defined as

8 $$\begin{aligned} \phi (\varpi )=\frac{\mu _1 e^{\sigma _1 \varpi }+\mu _2 e^{\sigma _2 \varpi }}{\mu _2 e^{\sigma _2 \varpi }+\mu _3 e^{\sigma _3 \varpi }}. \end{aligned}$$

**Step:3**

We apply the homogeneous balance technique on (7) to attain the value of N.

**Step:4** Substituting (7) with equation (8) into (6), then we attain the system of algebraic equations. The system is solved by utilizing Mathematica software, and then the achieved solution of (8) is put into (7) by using (5). Finally, the solution of (4) is attained.

## Solitary wave structure

We consider the travelling wave transformation for FDSW (2) as follows,

9 $$\begin{aligned} \Phi (x,t)=\Phi (\varpi ),~~\Psi (x,t)=\Psi (\varpi ),~~ \varpi =\kappa _1\left( x+\frac{\omega _1}{\alpha }(t+\frac{1}{\Gamma (\alpha )})^\alpha \right) . \end{aligned}$$

Using (9) to (2) and then we get ,

10 $$\begin{aligned}{} & {} a\kappa _1\Psi \Psi ^{'}-\kappa _1\omega _1\Phi ^{'}=0. \end{aligned}$$

11 $$\begin{aligned}{} & {} \lambda _1\kappa _1\Phi \Psi ^{'}+\varpi _{1}\kappa _1^3\Psi ^{'''}-\kappa _1\omega _1\Psi ^{'}+\Psi \gamma _1\kappa _1\Phi ^{'}=0. \end{aligned}$$

From (10), we have

12 $$\begin{aligned} \Phi =\frac{a \Psi ^2}{2\omega _1}. \end{aligned}$$

Putting the value of $\Phi$ into (11) and integrating one time then we get,

13 $$\begin{aligned} 6 \varpi _1 \kappa _1^2 \omega _1 \Psi ^{''}-6\omega _1^2\Psi +a(\lambda _1+2\gamma _1)\Psi ^3=0. \end{aligned}$$

Now we have to apply the balancing technique on (13) then we get $N=1$. Utilizing $N=1$ in (7) then we get,

14 $$\begin{aligned} \Psi (\varpi )=a_0+a_1\phi (\varpi )+b_1\phi (\varpi )^{-1}. \end{aligned}$$

where $a_0, a_1$, and $b_1$ are unknown constants to be find. The solution of (2) is discussed as,

**Case-1** If $\left[ \sigma _1, \sigma _2, \sigma _3 ,\sigma _4 \right]$=$[1, -1, 1, 1]$ and $[\mu _1, \mu _2, \mu _3, \mu _4]$=$[1, -1, 1, -1]$ then (8) become,

15 $$\begin{aligned} \phi (\varpi )=\textrm{Tanh}(\varpi ). \end{aligned}$$

When equations (14) and (15) are putting into equation (13), we arrive at a system of algebraic linear equations. By solving these equations simultaneously, we obtain the following set of solitary wave solutions. **set-1**

16 $$\begin{aligned} a_{0}= 0, b_{1}=-a_{1}, a_{1}=a_{1}, \gamma _{1}=\frac{-a a_{1}^{2} \lambda _{1}-48 \varpi _{1}^{2} \kappa _{1}^{4}}{2 a a_{1}^{2}}, \omega _{1}=4 \varpi _{1} \kappa _{1}^{2}. \end{aligned}$$

Putting (16) into (14) then solution of (2) is,

17 $$\begin{aligned} \Psi (\varpi )= & {} a_1 (-\text {csch}(\varpi )) \text {sech}(\varpi ),~~\varpi =\kappa _1\left( x+\frac{4 \varpi _1 \kappa _1^2}{\alpha }(t+\frac{1}{\Gamma (\alpha )})^\alpha \right) . \end{aligned}$$

18 $$\begin{aligned} \Phi (\varpi )= & {} \frac{a a_1^2 \text {csch}^2(2 \varpi )}{2 \varpi _1 \kappa _1^2},~~\varpi =\kappa _1\left( x+\frac{4 \varpi _1 \kappa _1^2}{\alpha }(t+\frac{1}{\Gamma (\alpha )})^\alpha \right) . \end{aligned}$$

**Set-2**

19 $$\begin{aligned} a_0= 0, b_1=a_1, a_1=a_1, \gamma _1=\frac{96 \varpi _1^2 \kappa _1^4-a a_1^2 \lambda _1}{2 a a_1^2}, \omega _1=-8 \varpi _1 \kappa _1^2. \end{aligned}$$

Substituting (19) into (14) then solution of (2) is,

20 $$\begin{aligned} \Psi (\varpi )= & {} a_1 (\tanh (\varpi )+\coth (\varpi ),~~\varpi =\kappa _1\left( x-\frac{8 \varpi _1 \kappa _1^2}{\alpha }(t+\frac{1}{\Gamma (\alpha )})^\alpha \right) . \end{aligned}$$

21 $$\begin{aligned} \Phi (\varpi )= & {} -\frac{a a_1^2 \left( \tanh (\varpi )+\coth (\varpi )\right) ^2}{16 \varpi _1 \kappa _1^2},~~\varpi =\kappa _1\left( x-\frac{8 \varpi _1 \kappa _1^2}{\alpha }(t+\frac{1}{\Gamma (\alpha )})^\alpha \right) . \end{aligned}$$

**Set-3**

22 $$\begin{aligned} a_0=0, a_1=a_1 b_1=0, \gamma _1=\frac{24 \varpi _1^2 \kappa _1^4-a a_1^2 \lambda _1}{2 a a_1^2}, \omega _1=-2 \varpi _1 \kappa _1^2. \end{aligned}$$

Putting (22) into (14) then solution of (2) is,

23 $$\begin{aligned} \Psi (\varpi )= & {} a_1\textrm{Tanh}(\varpi ),~~\varpi =\kappa _1\left( x-\frac{2 \varpi _1 \kappa _1^2}{\alpha }(t+\frac{1}{\Gamma (\alpha )})^\alpha \right) . \end{aligned}$$

24 $$\begin{aligned} \Phi (\varpi )= & {} -\frac{a a_1^2 \tanh ^2(\varpi )}{4 \varpi _1 \kappa _1^2},~~\varpi =\kappa _1\left( x-\frac{2 \varpi _1 \kappa _1^2}{\alpha }(t+\frac{1}{\Gamma (\alpha )})^\alpha \right) . \end{aligned}$$

**Set-4**

25 $$\begin{aligned} a_0=0, a_1=0, b_1=b_1, \gamma _1=\frac{24 \varpi _1^2 \kappa _1^4-a b_1^2 \lambda _1}{2 a b_1^2}, \omega _1=-2 \varpi _1 \kappa _1^2. \end{aligned}$$

Substituting (25) into (14) then solution of (2) is,

26 $$\begin{aligned} \Psi (\varpi )= & {} b_1\coth (\varpi ),~~\varpi =\kappa _1\left( x-\frac{2 \varpi _1 \kappa _1^2}{\alpha }(t+\frac{1}{\Gamma (\alpha )})^\alpha \right) . \end{aligned}$$

27 $$\begin{aligned} \Phi (\varpi )= & {} -\frac{a b_1^2 \coth ^2(\varpi )}{4 \varpi _1 \kappa _1^2},~~\varpi =\kappa _1\left( x-\frac{2 \varpi _1 \kappa _1^2}{\alpha }(t+\frac{1}{\Gamma (\alpha )})^\alpha \right) . \end{aligned}$$

**Case-2** If $\left[ \sigma _1, \sigma _2, \sigma _3 ,\sigma _4 \right] =[\imath , -\imath , 1, 1]$ and $[\mu _1, \mu _2, \mu _3, \mu _4]=[\imath , -\imath , \imath , -\imath ]$ then (8) become,

28 $$\begin{aligned} \phi (\varpi )=-\textrm{Tan}(\varpi ). \end{aligned}$$

When equations (28) and (15) are putting into equation (13), we arrive at a system of algebraic linear equations. By solving these equations simultaneously, we obtain the following set of solitary wave solutions.

**Set-1**

29 $$\begin{aligned} a_0= 0, b_1=-a_1, a_1=a_1, \gamma _1=-\frac{2 \left( a a_1^2 \gamma _1+48 \varpi _1^2 \kappa _1^4\right) }{a a_1^2}, \omega _1=8 \varpi _1 \kappa _1^2. \end{aligned}$$

Putting (29) into (14) then solution of (2) is,

30 $$\begin{aligned} \Psi (\varpi )= & {} a_1 \cos (2 \varpi ) \csc (\varpi ) \sec (\varpi ),~~\varpi =\kappa _1\left( x+\frac{8 \varpi _1 \kappa _1^2}{\alpha }(t+\frac{1}{\Gamma (\alpha )})^\alpha \right) . \end{aligned}$$

31 $$\begin{aligned} \Phi (\varpi )= & {} \frac{a a_1^2 \cot ^2(2 \varpi )}{4 \varpi _1 \kappa _1^2},~~\varpi =\kappa _1\left( x+\frac{8 \varpi _1 \kappa _1^2}{\alpha }(t+\frac{1}{\Gamma (\alpha )})^\alpha \right) . \end{aligned}$$

**Set-2**

32 $$\begin{aligned} a_0= 0, b_1=a_1, a_1=a_1, \gamma _1=-\frac{2 \left( a a_1^2 \gamma _1-24 \varpi _1^2 \kappa _1^4\right) }{a a_1^2}, \omega _1=-4 \varpi _1 \kappa _1^2. \end{aligned}$$

Substituting (32) into (14) then solution of (2) is,

33 $$\begin{aligned} \Psi (\varpi )= & {} a_1 (-\csc (\varpi )) \sec (\varpi ),~~\varpi =\kappa _1\left( x-\frac{4 \varpi _1 \kappa _1^2}{\alpha }(t+\frac{1}{\Gamma (\alpha )})^\alpha \right) . \end{aligned}$$

34 $$\begin{aligned} \Phi (\varpi )= & {} -\frac{a a_1^2 \csc ^2(2 \varpi )}{2 \varpi _1 \kappa _1^2},~~\varpi =\kappa _1\left( x-\frac{4 \varpi _1 \kappa _1^2}{\alpha }(t+\frac{1}{\Gamma (\alpha )})^\alpha \right) . \end{aligned}$$

**Set-3**

35 $$\begin{aligned} a_0=0, a_1=a_1, b_1=0, \gamma _1=-\frac{2 \left( a a_1^2 \gamma _1+12 \varpi _1^2 \kappa _1^4\right) }{a a_1^2}, \omega _1=2 \varpi _1 \kappa _1^2. \end{aligned}$$

Putting Eq. (35) into (14) then solution of (2) is,

36 $$\begin{aligned} \Psi (\varpi )= & {} -a_1\textrm{Tan}(\varpi ),~~\varpi =\kappa _1\left( x+\frac{2 \varpi _1 \kappa _1^2}{\alpha }(t+\frac{1}{\Gamma (\alpha )})^\alpha \right) . \end{aligned}$$

37 $$\begin{aligned} \Phi (\varpi )= & {} \frac{a \tan ^2(\varpi )}{4 \varpi _1 \kappa _1^2},~~\varpi =\kappa _1\left( x+\frac{2 \varpi _1 \kappa _1^2}{\alpha }(t+\frac{1}{\Gamma (\alpha )})^\alpha \right) . \end{aligned}$$

**Set-4**

38 $$\begin{aligned} a_0=0, a_1=0, b_1=b_1, \gamma _1=-\frac{2 \left( a b_1^2 \gamma _1+12 \varpi _1^2 \kappa _1^4\right) }{a b_1^2}, \omega _1=2 \varpi _1 \kappa _1^2. \end{aligned}$$

Substituting (38) into (14) then solution of (2) is,

39 $$\begin{aligned} \Psi (\varpi )= & {} b_1 (-\cot (\varpi )),~~\varpi =\kappa _1\left( x+\frac{2 \varpi _1 \kappa _1^2}{\alpha }(t+\frac{1}{\Gamma (\alpha )})^\alpha \right) . \end{aligned}$$

40 $$\begin{aligned} \Phi (\varpi )= & {} \frac{a b_1^2 \cot ^2(\varpi )}{4 \varpi _1 \kappa _1^2},~~\varpi =\kappa _1\left( x+\frac{2 \varpi _1 \kappa _1^2}{\alpha }(t+\frac{1}{\Gamma (\alpha )})^\alpha \right) . \end{aligned}$$

**Case-3** If $\left[ \sigma _1, \sigma _2, \sigma _3 ,\sigma _4 \right] =[1+\imath , 1-\imath , 1, 1]$ and $[\mu _1, \mu _2, \mu _3, \mu _4]=[\imath , -\imath , \imath , -\imath ]$ then (8) become,

41 $$\begin{aligned} \phi (\varpi )=1-\textrm{Tan}(\varpi ). \end{aligned}$$

When equations (41) and (15) are putting into equation (13), we arrive at a system of algebraic linear equations. By solving these equations simultaneously, we obtain the following set of solitary wave solutions.

**Set-1**

42 $$\begin{aligned} a_0=-a_1, a_1=a_1, b_1=0, \lambda _1=-\frac{2 \left( a a_1^2 \gamma _1+12 \varpi _1^2 \kappa _1^4\right) }{a a_1^2}, \omega _1=2 \varpi _1 \kappa _1^2. \end{aligned}$$

Putting (42) into (14) then solution of (2) is,

43 $$\begin{aligned} \Psi (\varpi )= & {} -a_1\textrm{Tan}(\varpi ),~~\varpi =\kappa _1\left( x+\frac{2 \varpi _1 \kappa _1^2}{\alpha }(t+\frac{1}{\Gamma (\alpha )})^\alpha \right) . \end{aligned}$$

44 $$\begin{aligned} \Phi (\varpi )= & {} \frac{a a_1^2 \tan ^2(\varpi )}{4 \varpi _1 \kappa _1^2},~~\varpi =\kappa _1\left( x+\frac{2 \varpi _1 \kappa _1^2}{\alpha }(t+\frac{1}{\Gamma (\alpha )})^\alpha \right) . \end{aligned}$$

**Case-4** If $\left[ \sigma _1, \sigma _2, \sigma _3 ,\sigma _4 \right] =[2+\imath , 2-\imath , 1, 1]$ and $[\mu _1, \mu _2, \mu _3, \mu _4]=[\imath , -\imath , \imath , -\imath ]$ then (8) become,

45 $$\begin{aligned} \phi (\varpi )=2+\textrm{Tan}(\varpi ). \end{aligned}$$

When equations (45) and (15) are putting into equation (13), we arrive at a system of algebraic linear equations. By solving these equations simultaneously, we obtain the following set of solitary wave solutions.

**Set-1**

46 $$\begin{aligned} a_0=-2 a_1, a_1=a_1, b_1=0, \lambda _1=-\frac{2 \left( a a_1^2 \gamma _1+12 \varpi _1^2 \kappa _1^4\right) }{a a_1^2}, \omega _1=2 \varpi _1 \kappa _1^2. \end{aligned}$$

Substituting (46) into (14) then solution of (2) is,

47 $$\begin{aligned} \Psi (\varpi )= & {} a_1\textrm{Tan}(\varpi ),~~\varpi =\kappa _1\left( x+\frac{2 \varpi _1 \kappa _1^2}{\alpha }(t+\frac{1}{\Gamma (\alpha )})^\alpha \right) . \end{aligned}$$

48 $$\begin{aligned} \Phi (\varpi )= & {} \frac{a a_1^2 \tan ^2(\varpi )}{4 \varpi _1 \kappa _1^2},~~\varpi =\kappa _1\left( x+\frac{2 \varpi _1 \kappa _1^2}{\alpha }(t+\frac{1}{\Gamma (\alpha )})^\alpha \right) . \end{aligned}$$

**Case-5** If $\left[ \sigma _1, \sigma _2, \sigma _3 ,\sigma _4 \right] =[2, 1, 1, 1]$ and $[\mu _1, \mu _2, \mu _3, \mu _4]=[1, 0, 1, 0]$ then (8) become,

49 $$\begin{aligned} \phi (\varpi )=\frac{2e^{\varpi }+1}{e^{\varpi }+1}. \end{aligned}$$

When equations (49) and (15) are putting into equation (13), we arrive at a system of algebraic linear equations. By solving these equations simultaneously, we obtain the following set of solitary wave solutions.

**Set-1**

50 $$\begin{aligned} a_0=-\frac{1}{4} \left( 3 b_1\right) , a_1= 0, b_1=b_1, \lambda _1=-\frac{2 \left( a b_1^2 \gamma _1-12 \varpi _1^2 \kappa _1^4\right) }{a b_1^2}, \omega _1=-\frac{1}{2} \varpi _1 \kappa _1^2. \end{aligned}$$

Putting (50) into (14) then solution of (2) is,

51 $$\begin{aligned} \Psi (\varpi )= & {} \frac{b_1 \left( 1-2 e^{\varpi }\right) }{8 e^{\varpi }+4},~~\varpi =\kappa _1\left( x-\frac{\varpi _1 \kappa _1^2}{2\alpha }(t+\frac{1}{\Gamma (\alpha )})^\alpha \right) . \end{aligned}$$

52 $$\begin{aligned} \Phi (\varpi )= & {} -\frac{a b_1^2 \left( 1-2 e^{\varpi }\right) ^2}{\left( 8 e^{\varpi }+4\right) ^2 \varpi _1 \kappa _1^2},~~\varpi =\kappa _1\left( x-\frac{\varpi _1 \kappa _1^2}{2\alpha }(t+\frac{1}{\Gamma (\alpha )})^\alpha \right) . \end{aligned}$$

**Set-2**

53 $$\begin{aligned} a_0=-\frac{1}{2} \left( 3 a_1\right) , b_1= 0, a_1=a_1, \lambda _1=-\frac{2 \left( a a_1^2 \gamma _1-3 \varpi _1^2 \kappa _1^4\right) }{a a_1^2}, \omega _1=-\frac{1}{2} \varpi _1 \kappa _1^2. \end{aligned}$$

Putting (53) into (14) then solution of (2) is,

54 $$\begin{aligned} \Psi (\varpi )= & {} \frac{a_1 \left( e^{\varpi }-1\right) }{2 \left( e^{\varpi }+1\right) },~~\varpi =\kappa _1\left( x-\frac{\varpi _1 \kappa _1^2}{2\alpha }(t+\frac{1}{\Gamma (\alpha )})^\alpha \right) . \end{aligned}$$

55 $$\begin{aligned} \Phi (\varpi )= & {} -\frac{a a_1^2 \left( e^{\varpi }-1\right) ^2}{4 \left( e^{\varpi }+1\right) ^2 \varpi _1 \kappa _1^2},~~\varpi =\kappa _1\left( x-\frac{\varpi _1 \kappa _1^2}{2\alpha }(t+\frac{1}{\Gamma (\alpha )})^\alpha \right) . \end{aligned}$$

**Case-6** If $\left[ \sigma _1, \sigma _2, \sigma _3 ,\sigma _4 \right] =[2, 0, 1, 1]$ and $[\mu _1, \mu _2, \mu _3, \mu _4]=[-1, 0, 1, -1]$ then (8) become,

56 $$\begin{aligned} \phi (\varpi )=1-\tanh (\varpi ). \end{aligned}$$

When equations (56) and (15) are putting into equation (13), we arrive at a system of algebraic linear equations. By solving these equations simultaneously, we obtain the following set of solitary wave solutions.

**Set-1**

57 $$\begin{aligned} a_0=-a_1, a_1=a_1, b_1=0, \lambda _1=-\frac{2 \left( a a_1^2 \gamma _1-12 \varpi _1^2 \kappa _1^4\right) }{a a_1^2}, \omega _1=-2 \varpi _1 \kappa _1^2. \end{aligned}$$

Putting (57) into (14) then solution of (2) is,

58 $$\begin{aligned} \Psi (\varpi )= & {} -a_1\textrm{Tanh}(\varpi ),~~\varpi =\kappa _1\left( x-\frac{2 \varpi _1 \kappa _1^2}{\alpha }(t+\frac{1}{\Gamma (\alpha )})^\alpha \right) . \end{aligned}$$

59 $$\begin{aligned} \Phi (\varpi )= & {} -\frac{a a_1^2 \tanh ^2(\varpi )}{4 \varpi _1 \kappa _1^2},~~\varpi =\kappa _1\left( x-\frac{2 \varpi _1 \kappa _1^2}{\alpha }(t+\frac{1}{\Gamma (\alpha )})^\alpha \right) . \end{aligned}$$

**Case-7** If $\left[ \sigma _1, \sigma _2, \sigma _3 ,\sigma _4 \right] =[-3, -1, -1, 1]$ and $[\mu _1, \mu _2, \mu _3, \mu _4]=[-1, 1, -1, 1]$ then (8) become,

60 $$\begin{aligned} \phi (\varpi )=\tanh (\varpi )-2. \end{aligned}$$

When equations (60) and (15) are putting into equation (13), we arrive at a system of algebraic linear equations. By solving these equations simultaneously, we obtain the following set of solitary wave solutions.

**Set-1**

61 $$\begin{aligned} a_0=2 a_1, a_1=a_1, b_1=0, \omega _1=-\frac{\sqrt{a} a_1 \sqrt{2 \gamma _1+\lambda _1}}{\sqrt{6}}, \varpi _1=\frac{\sqrt{a} a_1 \sqrt{2 \gamma _1+\lambda _1}}{2 \sqrt{6} \kappa _1^2}. \end{aligned}$$

Putting (61) into (14) then solution of (2) is,

62 $$\begin{aligned} \Psi (\varpi )= & {} a_1\textrm{Tanh}(\varpi ),~~\varpi =\kappa _1\left( x-\frac{\sqrt{a} a_1 \sqrt{2 \gamma _1+\lambda _1}}{\sqrt{6}\alpha }(t+\frac{1}{\Gamma (\alpha )})^\alpha \right) . \end{aligned}$$

63 $$\begin{aligned} \Phi (\varpi )= & {} -\frac{\sqrt{\frac{3}{2}} \sqrt{a} a_1 \tanh ^2(\varpi )}{\sqrt{2 \gamma _1+\lambda _1}},~~\varpi =\kappa _1\left( x-\frac{\sqrt{a} a_1 \sqrt{2 \gamma _1+\lambda _1}}{\sqrt{6}\alpha }(t+\frac{1}{\Gamma (\alpha )})^\alpha \right) . \end{aligned}$$

**Set-2**

64 $$\begin{aligned} a_0=2 a_1, a_1=a_1, b_1=0, \omega _1=\frac{\sqrt{a} a_1 \sqrt{2 \gamma _1+\lambda _1}}{\sqrt{6}}, \varpi _1=\frac{\sqrt{a} a_1 \sqrt{2 \gamma _1+\lambda _1}}{2 \sqrt{6} \kappa _1^2}. \end{aligned}$$

Putting (64) into (14) then solution of (2) is,

65 $$\begin{aligned} \Psi (\varpi )= & {} a_1\textrm{Tanh}(\varpi ),~~\varpi =\kappa _1\left( x+\frac{\sqrt{a} a_1 \sqrt{2 \gamma _1+\lambda _1}}{\sqrt{6}\alpha }(t+\frac{1}{\Gamma (\alpha )})^\alpha \right) . \end{aligned}$$

66 $$\begin{aligned} \Phi (\varpi )= & {} \frac{\sqrt{\frac{3}{2}} \sqrt{a} a_1 \tanh ^2(\varpi )}{\sqrt{2 \gamma _1+\lambda _1}},~~\varpi =\kappa _1\left( x+\frac{\sqrt{a} a_1 \sqrt{2 \gamma _1+\lambda _1}}{\sqrt{6}\alpha }(t+\frac{1}{\Gamma (\alpha )})^\alpha \right) . \end{aligned}$$

**Case-8** If $\left[ \sigma _1, \sigma _2, \sigma _3 ,\sigma _4 \right] =[1, 0, 1, 1]$ and $[\mu _1, \mu _2, \mu _3, \mu _4]=[0, 0, 1, 0]$ then (8) become,

67 $$\begin{aligned} \phi (\varpi )=\frac{1}{1+e^\varpi }. \end{aligned}$$

When equations (67) and (15) are putting into equation (13), we arrive at a system of algebraic linear equations. By solving these equations simultaneously, we obtain the following set of solitary wave solutions.

**Set-1**

68 $$\begin{aligned} a_0=a_0, a_1=-2 a_0, b_1=0, \omega _1=-\frac{\sqrt{a} a_0 \sqrt{2 \gamma _1+\lambda _1}}{\sqrt{6}}, \varpi _1=\frac{\sqrt{\frac{2}{3}} \sqrt{a} a_0 \sqrt{2 \gamma _1+\lambda _1}}{\kappa _1^2}. \end{aligned}$$

Putting (68) into (14) then solution of (2) is,

69 $$\begin{aligned} \Psi (\varpi )= & {} a_0(1-\frac{2}{1+e^\varpi }),~~\varpi =\kappa _1\left( x-\frac{\sqrt{a} a_1 \sqrt{2 \gamma _1+\lambda _1}}{\sqrt{6}\alpha }(t+\frac{1}{\Gamma (\alpha )})^\alpha \right) . \end{aligned}$$

70 $$\begin{aligned} \Phi (\varpi )= & {} -\frac{\sqrt{\frac{3}{2}} \sqrt{a} a_0 \left( 1-\frac{2}{e^{\varpi }+1}\right) ^2}{\sqrt{2 \gamma _1+\lambda _1}},~~\varpi =\kappa _1\left( x-\frac{\sqrt{a} a_0 \sqrt{2 \gamma _1+\lambda _1}}{\sqrt{6}\alpha }(t+\frac{1}{\Gamma (\alpha )})^\alpha \right) . \end{aligned}$$

**Set-2**

71 $$\begin{aligned} a_0=a_0, a_1=-2 a_0, b_1=0, \omega _1=\frac{\sqrt{a} a_0 \sqrt{2 \gamma _1+\lambda _1}}{\sqrt{6}}, \varpi _1=-\frac{\sqrt{\frac{2}{3}} \sqrt{a} a_0 \sqrt{2 \gamma _1+\lambda _1}}{\kappa _1^2}. \end{aligned}$$

Substituting (71) into (14) then solution of (2) is,

72 $$\begin{aligned} \Psi (\varpi )= & {} a_0(1-\frac{2}{1+e^\varpi }),~~\varpi =\kappa _1\left( x+\frac{\sqrt{a} a_0 \sqrt{2 \gamma _1+\lambda _1}}{\sqrt{6}\alpha }(t+\frac{1}{\Gamma (\alpha )})^\alpha \right) . \end{aligned}$$

73 $$\begin{aligned} \Phi (\varpi )= & {} \frac{\sqrt{\frac{3}{2}} \sqrt{a} a_0 \left( 1-\frac{2}{e^{\varpi }+1}\right) ^2}{\sqrt{2 \gamma _1+\lambda _1}},~~\varpi =\kappa _1\left( x+\frac{\sqrt{a} a_0 \sqrt{2 \gamma _1+\lambda _1}}{\sqrt{6}\alpha }(t+\frac{1}{\Gamma (\alpha )})^\alpha \right) . \end{aligned}$$

## Numerical simulation and discussion

**Figure 1:**
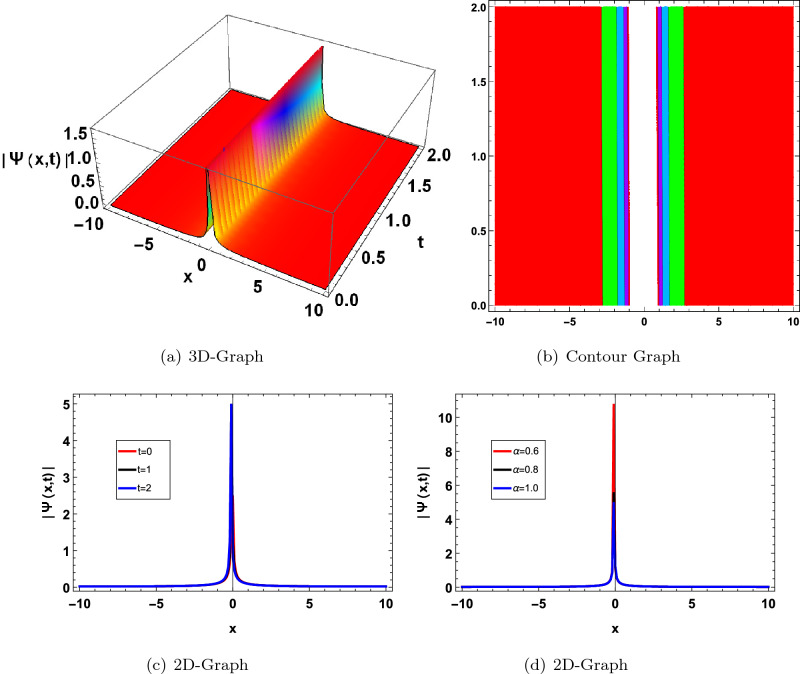
Graphical solution of (20) with parameters $\kappa _1 =-0.1, \varpi _1=-0.5, a_1=0.01$.

**Figure 2:**
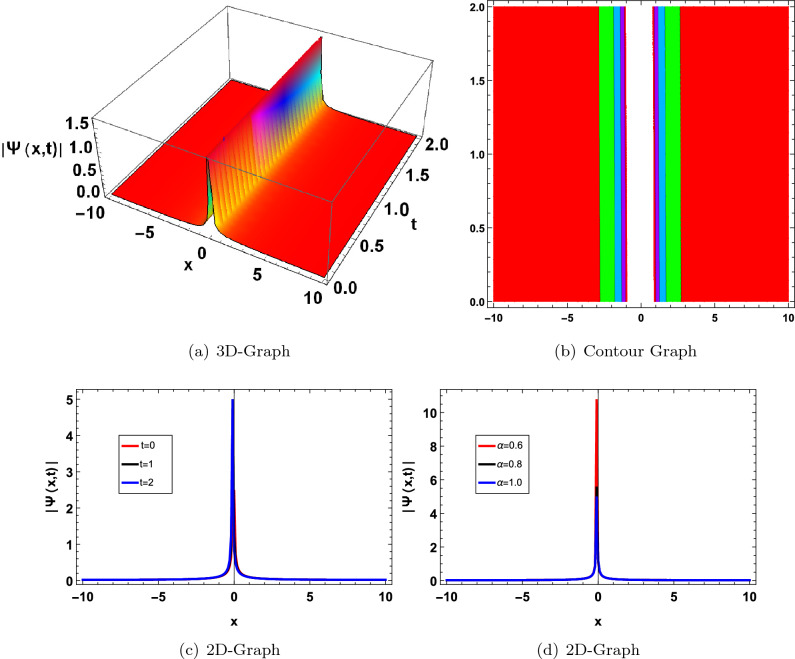
Graphical solution of (21) with parameters $\kappa _1=0.2, \varpi _1=-0.8, a_1=0.1, a=0.5$.

**Figure 3:**
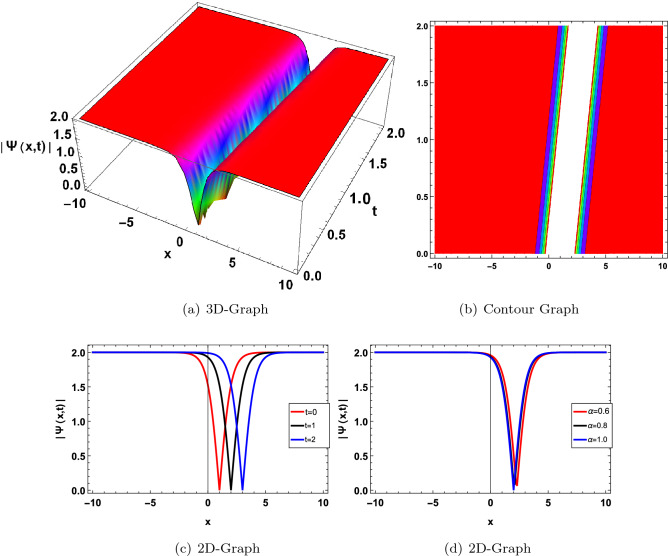
Graphical solution of (23) with parameters $\kappa _1=1, \varpi _1=0.5, a_1=2$.

**Figure 4:**
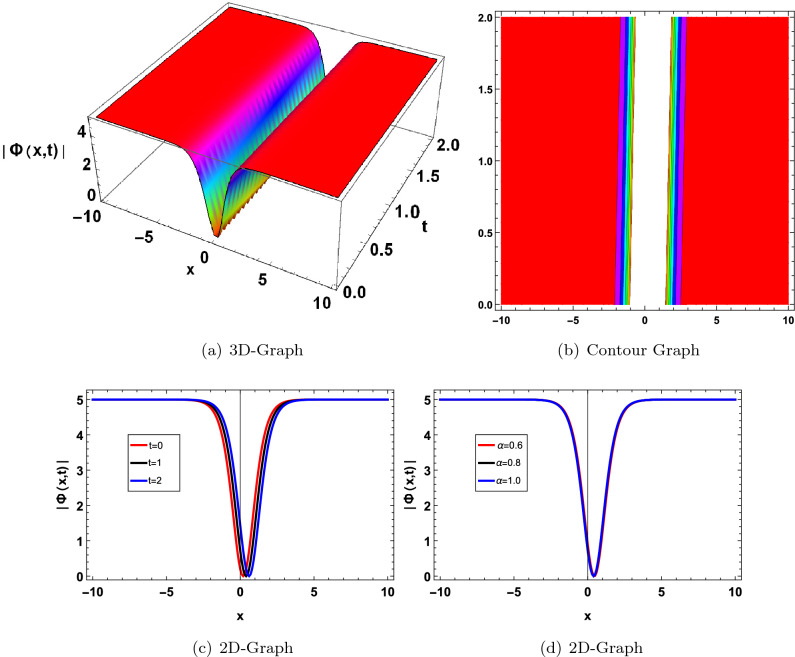
Graphical solution of (24) with parameters $\kappa _1 =1, \varpi _1 =0.1, a_1=1, a=2$.

**Figure 5:**
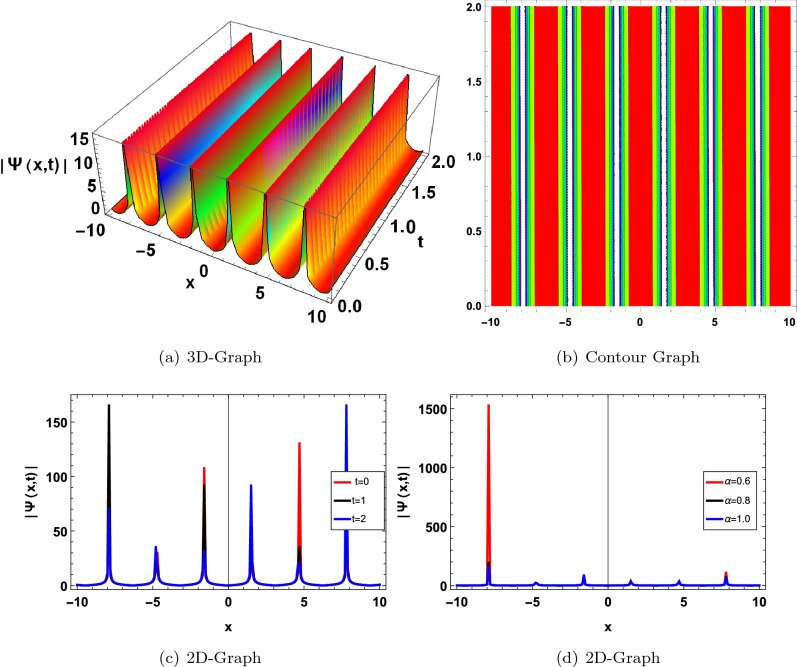
Graphical solution of (47) with parameters $\kappa _1=1, \varpi _1=0.01, a_1=1$.

**Figure 6:**
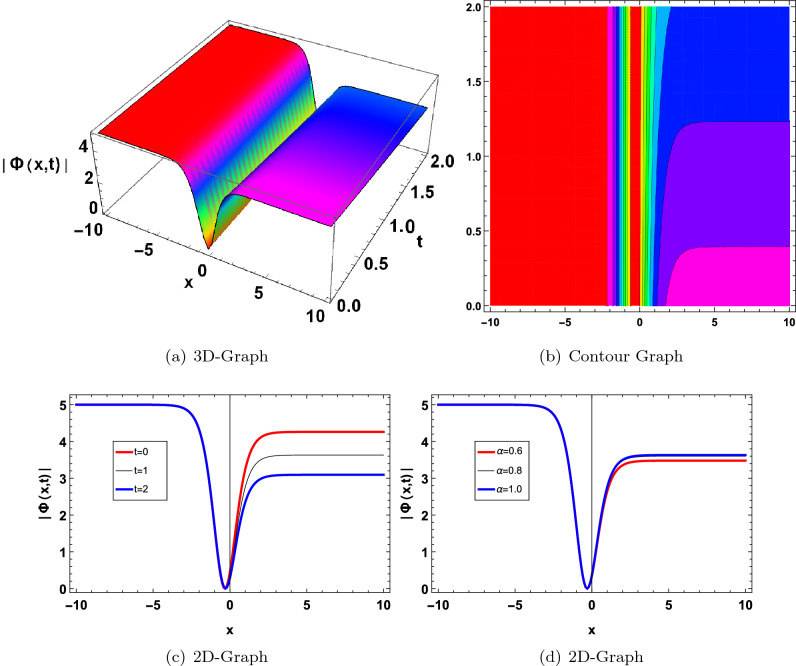
Graphical solution of (52) with parameters $\kappa _1=2, \varpi _1=0.01, b_1=2, a=0.8$.

**Figure 7:**
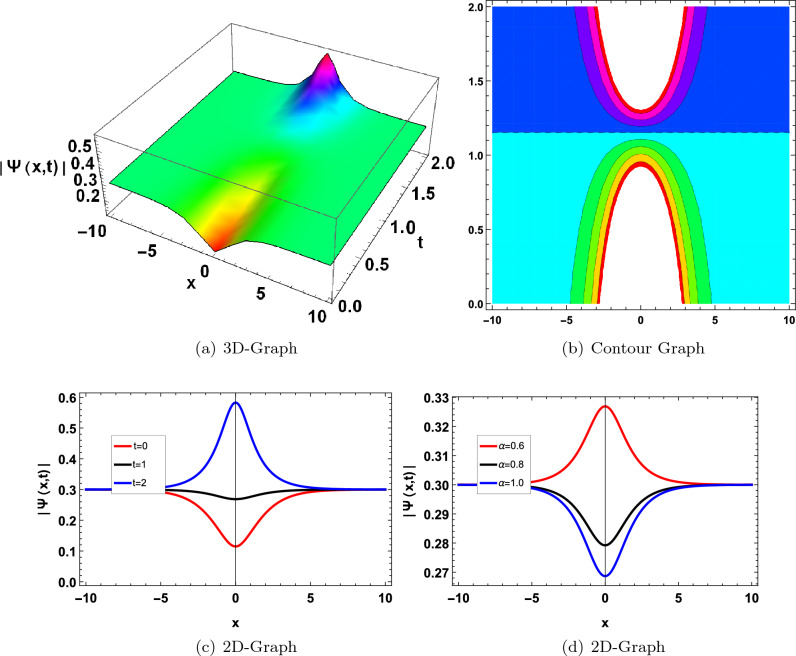
Graphical solution of (69) with parameters $\kappa _1=-0.8, \gamma _1=0.01, \lambda _1=0.02, a=-5, a_0=0.3, a_1=-5$.

**Figure 8:**
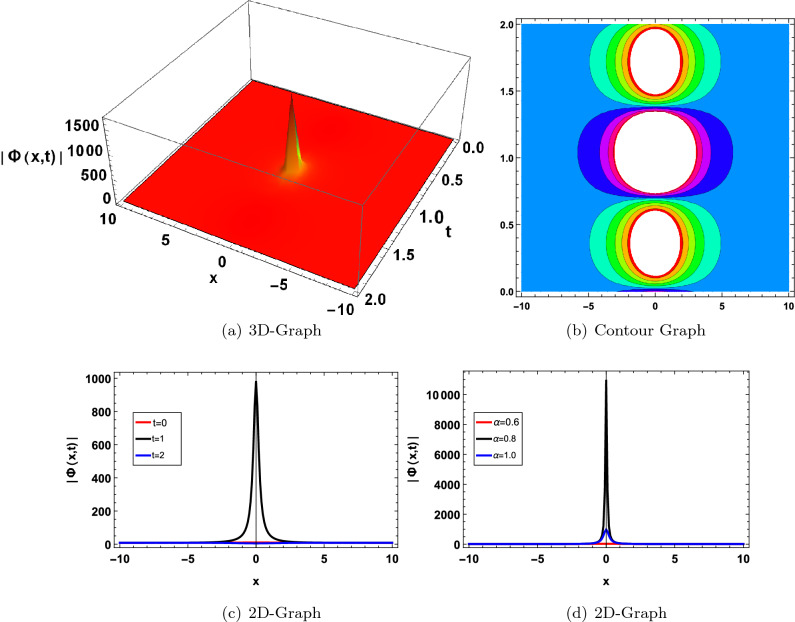
Graphical solution of (70) with parameters $\kappa _1=-0.8, \gamma _1=0.1, \lambda _1=0.2, a=-5, a_0=-2$.

In this section, we have drawn the graph of some attained solutions for the structure solution of solitary waves. The value fractional parameter $\alpha =1$ is fixed in all 2D graphs. Figs. (1 and 2) shows the singular bright soliton wave structure. Figures 3,4,6, 5, 7 and 8 shows the dark, periodic, bell and lump type soliton wave structure. In^59^ authors have attained the bright soliton solutions of the FDSW model by using the homotopy analysis transform method. Similarly in^60^ authors have achieved bright type soliton solution with the help of the Laplace Adomian decomposition method. Periodic-type soliton solutions have been attained by using the sine-cosine method^61^. But in this study, we get more generalized soliton solutions such as bright, dark, periodic, bell and lump.

## Modulus instability

We have found the modulation instability of the coupled nonlinear DSW model (1) through linear stability. We consider the steady-state solution,

74 $$\begin{aligned} \left\{ \begin{aligned} \Phi (x,t)= & {} \, \sqrt{P}+u(x,t) e^{P\delta \epsilon t}\\ \Psi (x,t)= & {} \, \sqrt{P}+v(x,t) e^{P\delta \epsilon t}. \end{aligned} \right. \end{aligned}$$

Substituting (74) into (1) then after linearize we get,

75 $$\begin{aligned} \left\{ \begin{aligned} u_{t}+P\delta \epsilon u+a\sqrt{P}v_{x}&=0 \\ v_{t}+P\delta \epsilon v+\gamma _1\sqrt{P} u_{x}+\lambda _1\sqrt{P}v_{x}+\varpi _1 v_{xxx}&=0. \end{aligned} \right. \end{aligned}$$

It is supposed that the solution of (75) has as,

76 $$\begin{aligned} \left\{ \begin{aligned} u(x,t)&=\rho _1 e^{\kappa x-\omega t}\\ v(x,t)&=\rho _2 e^{\kappa x-\omega t}, \end{aligned} \right. \end{aligned}$$

where $\kappa$ and $\omega$ are the wave number and frequency of perturbation. Putting (76) into (75), the dispersion relation (DR) is acquired as

77 $$\begin{aligned} \omega =\frac{\rho _2 \left( a \kappa \sqrt{P}+\varpi _1 \kappa ^3+\lambda _1+\delta P \epsilon \right) +\rho _1 \left( \gamma _1 \kappa \sqrt{P}+\delta P \epsilon \right) }{\rho _1+\rho _2}, \end{aligned}$$

from (77), one can see that the real component is negative for all values of $\kappa$ then any superposition of the results will appear to decay. So, the dispersion is stable.

## Conclusion

In this work, we have successfully achieved some fresh and further general traveling wave solutions to the nonlinear fractional Drinfel’d-Sokolov-Wilson (FDSW) model with beta derivative. The solutions attained by using the GERF method for the proposed model are competent to examine the scientific model of gravity water waves in shallow water. It is capable of investigating plasma waves in the seaside oceans and breaking down the unidirectional spread of long waves in oceans and harbors. The proposed method is not only more powerful than previous approaches but has also introduced novel solutions that have not been reported before.

## Data Availability

All data that support the findings of this study are included in the article.
